# Induced Gamma-Band Activity during Actual and Imaginary Movements: EEG Analysis

**DOI:** 10.3390/s20061545

**Published:** 2020-03-11

**Authors:** Carlos Amo Usanos, Luciano Boquete, Luis de Santiago, Rafael Barea Navarro, Carlo Cavaliere

**Affiliations:** Biomedical Engineering Group. Department of Electronics, Universidad de Alcalá. Plaza de San Diego, s/n 28801 Alcalá de Henares, Spain; caumtsz@gmail.com (C.A.U.); luis.san.ro@gmail.com (L.d.S.); rafael.barea@uah.es (R.B.N.); carlo.cavailere@uah.es (C.C.)

**Keywords:** electroencephalography, gamma-band activity, motor areas, imaginary motor tasks, actual motor tasks, event-related synchronization, power spectral density

## Abstract

The purpose of this paper is to record and analyze induced gamma-band activity (GBA) (30–60 Hz) in cerebral motor areas during imaginary movement and to compare it quantitatively with activity recorded in the same areas during actual movement using a simplified electroencephalogram (EEG). Brain activity (basal activity, imaginary motor task and actual motor task) is obtained from 12 healthy volunteer subjects using an EEG (Cz channel). GBA is analyzed using the mean power spectral density (PSD) value. Event-related synchronization (ERS) is calculated from the PSD values of the basal GBA (GBAb), the GBA of the imaginary movement (GBAim) and the GBA of the actual movement (GBAac). The mean GBAim and GBAac values for the right and left hands are significantly higher than the GBAb value (p = 0.007). No significant difference is detected between mean GBA values during the imaginary and actual movement (p = 0.242). The mean ERS values for the imaginary movement (ERSimM (%) = 23.52) and for the actual movement (ERSacM = 27.47) do not present any significant difference (p = 0.117). We demonstrated that ERS could provide a useful way of indirectly checking the function of neuronal motor circuits activated by voluntary movement, both imaginary and actual. These results, as a proof of concept, could be applied to physiology studies, brain–computer interfaces, and diagnosis of cognitive or motor pathologies.

## 1. Introduction

The synchronization of neuronal firing in the 20–200 Hz range is known as gamma-band activity (GBA) and can be divided into two bands, low (30–60 Hz) and high (60–200 Hz) [1,2]. GBA is generated in most brain structures, at a retinal level, and in the olfactory system. The principal neurotransmitters involved in its generation are glutamate (excitatory), acetylcholine and gamma-aminobutyric acid (inhibitory); GBA is linked to cerebral functions such as perception, attention, memory, consciousness, synaptic plasticity and motor control [3].

Neurophysiological studies have documented that in subjects at rest or performing motor tasks GBA in the 30–90 Hz frequency range appears in extensive areas of the brain [4]. Furthermore, movement-related GBA has been proposed as the integrator of sensory and motor processes during movement preparation and control [5].

GBA in voluntary movement (actual or imaginary) can be evaluated using intracranial electrodes [6] or electrocorticography (ECoG) [7,8,9,10,11]. These invasive methods, however, are largely inapplicable in standard clinical practice.

Several papers describe GBA recorded using non-invasive, low-cost and easily accessible methods such as conventional surface electroencephalograms (EEGs). The evaluation of activity in the gamma band using EEG records for actual, but not imaginary movements, was investigated in [12,13,14,15,16,17]. 

Motor imagery may be defined as a dynamic state during which representations of a given motor act are internally rehearsed in working memory without any motor output [18]. In this mental task subjects are instructed to imagine themselves moving without performing that movement so without muscle activation. The available evidence indicates that actual and imaginary movements share a substantial overlap of common functional circuits [19].

Previous papers describe the analysis of the GBA obtained during an imaginary movement from EEG recordings employing invasive methods (ECoG) [11,20]. 

Advances in obtaining GBA non-invasively during imaginary movement using conventional EEGs are presented in Table 1. These papers analyze various frequency intervals, use differing imaginary movement paradigms and employ a high number of EEG channels.

Acquiring the EEG signal in the gamma band is problematic. The EEG signal is generally contaminated by interferences from physiological signals (electrocardiogram (ECG), electromyogram (EMG), electrooculogram (EOG), etc.) and non-physiological artifacts (power line noise, electronic devices, etc.) [27]. Moreover, due to the 1/f^n^ nature of the EEG spectra, the decrease in power with the increase in frequency [28] makes it more difficult to obtain responses in the gamma band than in other lower EEG frequencies. 

Multichannel EEG systems make it possible to obtain spatial resolutions and apply signal separation algorithms, such as Independent Component Analysis (ICA) [29], so as to obtain responses in the gamma band. However, the setup process is tedious (attaching the electrodes, adjusting the impedance) and participants find the system uncomfortable. In recent years, research has been conducted into use of monochannel EEG systems in brain–computer interfaces (BCI´s) [30] (analyzed band: 0.5–10 Hz), [31] (steady-state visual evoked potentials), sleep studies [32,33], etc. However, to our knowledge, a single-channel system for detecting activity in the gamma band has not been implemented in imaginary movements.

The authors of this paper hypothesize that it is possible to analyze EEG activity in the gamma band during both actual and imaginary movements using a simple and quantifiable method easily applicable in daily practice.

Possible applications of a system with these characteristics may be the implementation of BCIs or in the study of diseases that affect cognitive or motor functions.

The advantage of a BCI can be focused on the implementation of a communication system [34], real-time control of peripherals as robots [35,36,37] or emotion recognition, among other. Many of the current BCI based on the detection of EEG imaginary movements (for a review [38]) analyze the alpha/beta EEG band, usually using several channels. The success of motor imagery BCI in translational applications is established in three learning pillars: at the machine, subject, and application level [39]. Some BCI´s multichannel works that analyze the gamma band can be referenced: [40] (19 EEG channels) [41] (21 channels), or [42] (128 channels).

Cognitive [43] and motor [44] functions affect the activity of the gamma band; consequently, a single-channel EEG system may be interesting for clinical use in the study of Alzheimer’s disease [45,46], depression [47], schizophrenia [48], etc. and also in some cortical diseases (traumatic vascular pathology and degenerative lesions).

The primary purpose of this paper is to obtain and analyze gamma activity in the 30–60 Hz frequency range caused by motor activity using a simplified EEG recording taken while performing an imaginary and actual motor task. The secondary purpose is to compare that activity during the imaginary motor task with the GBA obtained during the actual motor task.

## 2. Material and Methods

### 2.1. Participants and Experiment Description

All subjects were over the age of 18, have been informed about the details of the investigation and signed the informed consent according to a protocol approved by the local ethics committees of the University of Alcalá (Spain) and compliant with the tenets of the Declaration of Helsinki.

The study cohort for this experiment comprised 12 subjects (3 females and 9 males; mean age = 28.7; range = 21–47). All sample subjects were healthy and free of medical, neurological (including craniocerebral trauma and epilepsy) and psychiatric disease. None of the subjects were taking medicinal products and none had a record of alcohol or drug abuse or dependency.

According to the Edinburgh Handedness Inventory [49] were identified 9 right-handed subjects, 2 left-handed subjects, and 1 ambidextrous subject.

Details of the methodology employed in the experiment and in acquisition and analysis of the data in the EEG recordings have been published previously [17]. In brief, each subject is seated facing a computer monitor. They are positioned at a distance of 0.8 m and rest their forearms on a table with the palms of their hands facing downward. The experiment comprises 3 steps:

1. *Basal recording*. Participants keep their eyes fixed on the center of the screen (to prevent eye movement; they also try not to blink) and refrain from performing any motor or specific mental activity. A total of 18 min of basal activity are recorded, divided into 3 parts (each 6 min long) with a rest of approximately 1 min between each. This step is the first performed by the participants.
2. *Imaginary motor task.* An on-screen cue triggers the imaginary motor task, thereby obtaining in the EEG trace the motor GBA induced by that imaginary movement. The imaginary task consists of simulating, without muscle activation, rapid extension of the wrist followed by brief relaxation. This phase lasts approximately 40 min.
3. *Actual motor task.* The subjects perform an actual motor task with the same characteristics and duration of imaginary motor task.

During the motor activities, the physical conditions for the participants were the same as in the basal stage, the only difference being that they performed the actual or imaginary activities. Each trial lasted 2 s and started (at t = 0 s) with display (for 150 ms) of the cue in the center of the computer monitor. This was followed by a white screen that remained in place until the start of the next trial (t = 2 s). The motor experiments comprised 5 runs per hand alternated between right and left to prevent mental and muscle fatigue and each run comprised 100 trials. For both the imaginary motor task and the actual motor task, the subjects practiced the exercise in a training session held before the experiment was conducted.

### 2.2. Data Acquisition

The EEG signals were acquired with a 32-channel Micromed EEG (Handy EEG SD32) and the SystemPlus Evolution (Micromed SpA, Treviso, Italy) software, using a 2048-Hz sampling frequency, band-pass filters from 0.15–537.53 Hz and a 50-Hz notch filter. Electrode impedance was below 10 kΩ. The experiment was performed in a conventional laboratory with lights turned off and rechargeable batteries were used in the acquisition equipment to minimize potential alternating current induction at 50 Hz in the EEG power cables [50].

The EEG (active electrodes (C3, C4, Cz), reference (FPz), earth (Pz)) was recorded in continuous mode using Ag/AgCl electrodes. The electrooculogram was recorded to monitor eye movements. The electromyogram was obtained using two surface electrodes (active and reference) above the extensor carpi radialis longus muscle to confirm the movement in the actual motor task and the lack of movement in the imaginary motor task.

### 2.3. Data Analysis

The electrophysiological data were analyzed using MATLAB R2017b (The MathWorks Inc. Natick, MA, USA) and FieldTrip [51].

For analysis of both the basal EEG and during the motor tasks, 2-s segments corresponding to the trials established in the experiment were used. Band-pass (1 Hz, 100 Hz) and notch filters (band eliminated: 49–51 Hz) were applied. FieldTrip functions were used to complete signal processing, obtaining an artifact-free signal that was averaged for each subject and each hand. Finally, all the subjects were averaged to produce a grand average.

The GBA was quantified as spectral power values for the frequency band (low gamma band: 30–60 Hz, according to the taxonomy defined in [1]) using a multitaper Fast-Fourier transform (FieldTrip ft_freqanalysis function).

To analyze the GBA, only the Cz channel signals were used, as centrally channels were the least contaminated by movement and EMG artifacts [52]. Moreover, analyzing one of the central channels ensures that the implemented system does not depend on a subject’s hand dominance since it is known that the answer during a motor imagery task differs according to handedness [53,54].

### 2.4. Calculation of the GBA

The results for the GBA were expressed as the mean power spectral density (PSD) value in µV^2^. Based on the mean PSD values, the following parameters were defined:

- GBA during the basal experiment: GBAb.
- GBA during actual motor tasks: GBAac.
- GBA during imaginary motor tasks: GBAim.

The corresponding suffix was added to indicate right hand, left hand or mean of both (-R, -L, -M). For example: GBAacR indicates the GBA obtained from the actual movement of the right hand; GBAimL indicates the GBA obtained from the imaginary movement of the left hand; and GBAimM indicates the mean of the GBA obtained from the imaginary movement of the right and left hands.

### 2.5. Calculation of ERS for the GBA

Quantification of ERS in imaginary or actual movements was defined as a power increase relative to the basal state (GBAb). For this purpose, the GBA values of the motor tasks were normalized relative to the basal activity and expressed as a percentage [55]. For example, Equations (1) and (2) represent the ERS values for the means of both hands for the actual movement (ERSacM) and the imaginary movement (ERSimM).

(1) $$\mathrm{ERSacM}\left(\%\right)\;=\;\frac{\mathrm{GBAacM}-\mathrm{GBAb}}{\mathrm{GBAb}}\;\times 100\;$$

(2) $$\mathrm{ERSimM}\left(\%\right)\;=\;\frac{\mathrm{GBAimM}\;-\;\mathrm{GBAb}}{\mathrm{GBAb}}\;\times 100\;$$

Positive ERS values indicate a power increase in the activity compared to the basal situation. Both the GBA and the ERS were calculated for each trial and averaged for each hand. The corresponding suffix was added to indicate right hand, left hand or mean of both (-R, -L, -M). The grand average was then calculated for all the subjects. Finally, the mean ERS values for the actual movement (ERSacM) were compared with the mean values for the imaginary movement (ERSimM). 

### 2.6. Statistical Analysis

Statistical tests were performed using the SPSS 25.0 software (SPSS Inc. Chicago, IL, USA). Normally distributed variables are expressed as mean ± standard deviation; non-normally distributed variables are reported as median (interquartile range [IQR]). 

The normality of the results was assessed using the Shapiro–Wilk (W) test. The results were compared using the dependent t-test (paired-samples t-test) in normal distributions or the Wilcoxon signed-rank test (Z) in non-normal distributions. A p value below 0.05 was considered statistically significant.

## 3. Results 

The results of the study are shown in the tables below as the mean of the values for the entire sample (12 subjects), for each hand and both hands.

**Figure 1:**
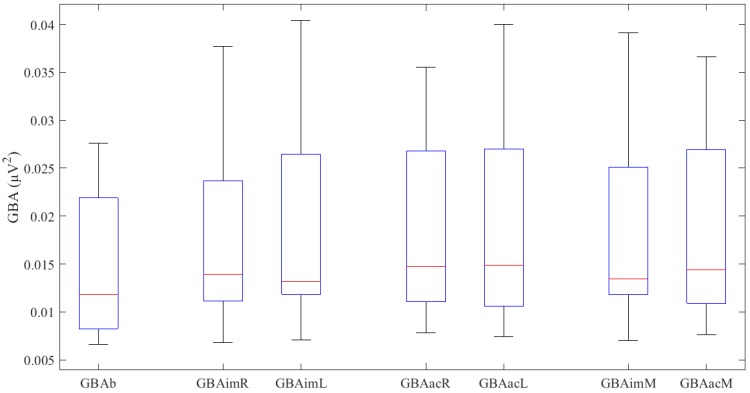
GBA values (μV^2^) obtained in the various experiments. The upper and lower box ends denote the first and third quartiles; the red line represents the median, and the whiskers represent 1.5 x interquartile range.

First, the results for basal activity (GBAb) were obtained, followed by those for the imaginary motor task (GBAim) and those for the actual motor task (GBAac). The latter two results are expressed as right hand, left hand, and mean for both hands. Figure 1 shows the results obtained in a box plot format. All the GBA values obtained (Table 2) follow a normal distribution, except GBAimL (W = 0.86, p = 0.049). No significant differences were found between imaginary and actual activity in either the right hand (p = 0.237), the left hand (p = 0.783) or the mean (p = 0.242).

The basal GBA activity is significantly lower relative to the imaginary movement of the right hand (t (11) = −3.127, p = 0.010), the left hand (Z = −3.059, p = 0.002) and the mean (t (11) = −3.321, p = 0.007). We also found that the basal GBA is significantly lower relative to the actual movement of the right hand (t (11) = −5.493, p = 0.0001), the left hand (t (11) = −3.752, p = 0.003) and the mean (t (11) = −4.965, p = 0.0001).

**Figure 2:**
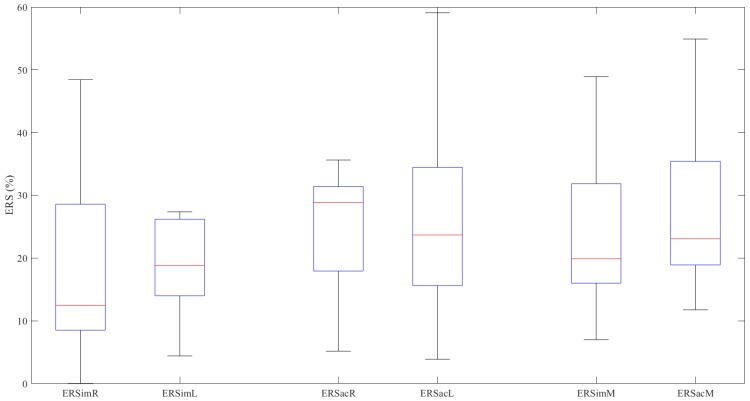
ERS values (%) in the various experiments.

Figure 2 represents the ERS values graphically. The ERSacL (W = 0.895, p = 0.138) and ERSacM (W = 0.863, p = 0.054) values follow a normal distribution; the other results do not meet the condition of normality (*p* < 0.044 in all cases).

The Wilcoxon signed-rank test finds no significant difference between the ERS values obtained in the imaginary movements and those obtained in the actual movements for either the right hand (Z = −1.020, p = 0.308), the left hand (Z = 0.471, p = 0.638) or the mean values (Z = −1.569, p = 0.117) (Table 3).

In the imaginary movements (ERS), there is no significant difference between right- and left-hand data distributions (Z = −1.726, p = 0.084). Neither is there any significant difference between the activity (ERS) for the right and left hands in the actual movements (Z = −0.235, p = 0.814).

In conclusion, our results indicate a significant increase in GBA, relative to basal activity, in both the actual movements and the imaginary movements for both hands. Furthermore, there is no significant difference between the imaginary and actual movements in both hands.

## 4. Discussion

The purpose of this paper has been to obtain and analyze the GBA in the cerebral motor areas by taking a monochannel EEG recording during an imaginary motor task and comparing that recording with the GBA obtained during an actual motor task. In this experiment, the basal GBA is significantly lower than the GBA values obtained during the imaginary and actual movements. Furthermore, no significant differences (p = 0.117) are observed between the ERS values for the actual and imaginary movements (ERSimM ≈ ERSacM). 

No previous papers analyze GBA using a combination of a single-channel (Cz) EEG and our stimulation and analysis frequency paradigm. Obtaining GBA during imaginary motor tasks using a conventional 64-channel EEG is described in [21], who analyze the 32–48 Hz EEG band, intending to discriminate wrist movement imagery. The purpose of the paper is to implement a brain–computer interface and it does not report ERS values. In a study by [22], greater GBA activity is observed during the imaginary movement than during the actual movement. This contrasts with our results, in which we found greater activity in the actual movement (ERSacM (%) = 27.479 ± 13.256, ERSimM (%) = 15.983 (14.313), with no significant difference). This contradiction may be due to the use of different types of movement, a different number of EEG channels and analysis of different gamma-band frequencies.

As is the case in our study, [23] observe significant increases in the power of the GBA during finger movement imagery. However, it does not report numerical values that would make it possible to compare results.

In [25] the authors observe that actual and imagery movements active sensorimotor and associative areas of both hemispheres, especially at 52–70 Hz band. They also found differences in activity between the actual movement and the imaginary movement in the frequencies analyzed (30–48 Hz: p = 0.000; 52–70 Hz: p = 0.011) in the temporal–parietal–occipital zone.

Works [24,26] likewise describe an increase, relative to basal activity, in the gamma band in both the imaginary movement and the actual movement. In addition, they do not find any difference in GBA between the actual movement and the imaginary movement. These papers, however, do not report overall numerical data for GBA since they focus on its anatomical location. In our case, the results express PSD values in the form of ERS to simplify their quantification for clinical purposes.

As regards the GBA obtained during imaginary movement using invasive methods [11,20], the results are not comparable with ours because in these previous studies they were obtained directly from the cerebral cortex (ECoG) without attenuation of the signal-to-noise ratio or the muscle artifacts found in a conventional EEG.

The GBA values we obtained during actual movement, however, are comparable with those of previous papers, the results of which are as follows: ERS ≈ 10–20 % [13,14] and ERS ≈ 20–30 % [17] and have values similar to ours (ERS ≈ 20–28 %).

Based on our study, it can be concluded that the ERS values for the imaginary and actual tasks do not show any significant difference (ERSimM ≈ ERSacM, (*p* > 0.05)). Activation of the same cortical areas during the actual and imaginary movements has been demonstrated in previous papers. The motor imagery belongs to the same category of processes involved in the programming and preparation of actual actions, the difference being that in this latter case, execution would be blocked at the corticospinal level. It can be assumed that the motor imagery shares the same neuronal mechanisms responsible for preparing and programming actual movements [18]. This hypothesis can be confirmed by experimentation e.g., using neuroimaging techniques to map cerebral activity during the imaginary movements, which reveals an activation pattern similar to that of execution of an actual action [56]. During the imaginary movement of the hand, there is an increase in the power of the gamma bands relative to the resting state (ERS), producing a significant overlap in spatial distribution (cortical areas) with the actual movement [11].

## 5. Conclusions

In this paper, we have developed a proof of concept that could confirm the viability of detecting gamma-band activity in imaginary and actual motor movements in environments compatible with clinical practice, doing so using a single EEG channel and without the need for a shielded chamber room.

Possible improvements to this experiment could include increasing the number of subjects and making the sample more homogeneous in terms of age and manual laterality (e.g., recruiting equal numbers of right-handed, left-handed, and ambidextrous subjects). It would also be beneficial to instruct subjects to close their eyes during the experiment and use an auditory stimulus or a non-cued paradigm (self-paced condition), making continual hand movements to avoid blinking artifacts.

At signal processing level, it would be convenient to implement some method for physiological artifact identification and removal in EEG registers (see [57] for a review). Consequently, it is intended to evaluate the detection capacity of GBA using artifact reduction techniques in single-channel acquisition systems, designed to eliminate some particular type of interference (e.g., ocular movements [58]) or more generalists ones [59].

This paper shows a proof of concept that explains the way to extract the gamma-band activity by a simple motor experiment (real or imaginary). It is not a method to discriminate between GBAim and GBAac, nor between the anatomical origin of the GBA (right or left hemisphere). However, this method could be used to create protocols applicable to BCI´s that can take advantage of both GBAim and GBAac, as in the distinction between imaginary movements of hands versus feet. Moreover, more variety of BCI codes could be created using the GBA signal obtained from both cerebral hemispheres.

If the results of this paper were confirmed in more exhaustive studies, gamma-band detection of imaginary movements could be used in the implementation of BCI´s, supporting the evaluation of cognitive functions in some cortical diseases (traumatic vascular pathology and degenerative lesions) or for use in assessing the pathology of motor areas, following up rehabilitation processes.

## Figures and Tables

**Table 1:** Studies analyzing GBA non-invasively during imaginary movement using conventional EEGs.

Authors	Number of Subjects	Channels	Frequency Range	Imagined Movement	Main Conclusions
Khan and Sepulveda, (2010) [21]	5	64	32–48 Hz	Wrist: extension, flexion, pronation, and supination.	An average recognition rate of approximately 89% was achieved in four movement types between the left and right wrists.
Kiroi et al., (2012) [22]	8	14	31–45 Hz 55–70 Hz	Flexion or oscillatory movement of the arm at the elbow, clenching of the hand.	Increase in activation levels, particularly in the central areas of the cortex.
Smith et al., (2014) [23]	10	54	70–150 Hz	Finger movement imagery.	Significant power increase was observed during motor imagery.
Korik et al., (2018) [24]	12	41	28–40 Hz	Imagined 3D limb movement.	The power spectral density contributes to the encoding of movement-related information during arm movement.
Lazurenko et al., (2018) [25]	24	17	30–48 Hz and 52–70 Hz	Imaginary hand, leg, and tongue movements.	Sensorimotor and associative areas of both hemispheres were actively involved in imaginary and actual movements.
Veslin et al., (2019) [26]	12	14	35–45	Right and left elbow movements.	Similar activity was obtained in the gamma band during the preparation and execution of both actual and imaginary movements.

**Table 2:** Analysis of the GBA data obtained.

Action	GBA	μV^2^	Comparison of Means
Basal	GBAb *	0.0145 ± 0.0076	-----
Right Hand	GBAimR *	0.0175 ± 0.0098	t(11) = −1.251, p = 0.237
	GBAacR *	0.0185 ± 0.0097	
Left Hand	GBAimL	0.0131 (0.0159)	Z = 0.275, p = 0.783
	GBAacL *	0.0185 ± 0.0104	
Mean Values	GBAimM *	0.0180 ± 0.0101	t(11) = 1.236, p = 0.242
	GBAacM *	0.0185 ± 0.0099	

* Normal distribution, Shapiro–Wilk test (*p* > 0.05).

**Table 3:** Analysis of the ERS data obtained.

	ERS	ERS (%)	Comparison of Means Wilcoxon Signed-Rank Test
Right Hand	ERSimR	12.435 (21.124)	Z = −1.020, p = 0.308
	ERSacR	28.850 (14.889)	
Left Hand	ERSimL	18.828 (13.578)	Z = −0.471, p = 0.638
	ERSacL *	26.972 ± 17.447	
Mean Values	ERSimM	15.983 (14.313)	Z = −1.569, p = 0.117
	ERSacM *	27.479 ± 13.256	

* Normal distribution, Shapiro–Wilk test (*p* > 0.05).
